# Assessment of Predictive Scoring System for 90-Day Mortality Among Patients With Locally Advanced Head and Neck Squamous Cell Carcinoma Who Have Completed Concurrent Chemoradiotherapy

**DOI:** 10.1001/jamanetworkopen.2019.20671

**Published:** 2020-03-26

**Authors:** Kuan-Chou Lin, Tsung-Ming Chen, Kevin Sheng-Po Yuan, Alexander T. H. Wu, Szu-Yuan Wu

**Affiliations:** 1 Department of Oral and Maxillofacial Surgery, Wan Fang Hospital, Taipei Medical University, Taipei, Taiwan; 2 Department of Otorhinolaryngology, Shuang-Ho Hospital, Taipei Medical University, Taipei, Taiwan; 3 Department of Otorhinolaryngology, Wan Fang Hospital, Taipei Medical University, Taipei, Taiwan; 4 Program for Translational Medicine, Taipei Medical University, Taipei, Taiwan; 5 Department of Food Nutrition and Health Biotechnology, Asia University College of Medical and Health Science, Taichung, Taiwan; 6 Division of Radiation Oncology, Lo-Hsu Medical Foundation, Lotung Poh-Ai Hospital, Yilan, Taiwan; 7 Big Data Center, Lo-Hsu Medical Foundation, Lotung Poh-Ai Hospital, Yilan, Taiwan; 8 Department of Healthcare Administration, College of Medical and Health Science, Asia University, Taichung, Taiwan

## Abstract

**Importance:**

There is currently no system to predict 90-day morality among patients with locally advanced head and neck squamous cell carcinoma (HNSCC) after the completion of concurrent chemoradiotherapy (CCRT).

**Objective:**

To validate the accuracy of a predictive scoring system for 90-day mortality among patients with locally advanced HNSCC who have completed CCRT.

**Design, Setting, and Participants:**

This prognostic study included 16 029 patients with HNSCC who completed CCRT between January 2006 and December 2015. Data were extracted from the Taiwan Cancer Registry Database. A risk scoring system was developed based on significant risk factors and corresponding risk coefficients. Data analysis was conducted from June 2018 to February 2019.

**Exposures:**

Mortality within 90 days of completion of definitive CCRT.

**Main Outcomes and Measures:**

The 90-day mortality rate after completion of CCRT and the accuracy of the scoring system, based on a comparison of mortality rates between training and test data sets.

**Results:**

Among 16 029 patients with locally advanced HNSCC, 1068 (6.66%; 1016 [95.1%] men; mean [SD] age, 55.11 [11.45] years) died before reaching the 90-day threshold, and 14 961 (93.4%; 14 080 [94.1%] men; mean [SD] age, 52.07 [9.99] years) survived. Multivariable analysis revealed that being aged 50 years or older (adjusted hazard ratio [aHR], 1.263; 95% CI, 1.104-1.445; *P* < .001), being aged 70 years or older (aHR, 2.183; 95% CI, 1.801-2.645; *P* < .001), having pneumonia (aHR, 1.946; 95% CI, 1.636-2.314; *P* < .001), having sepsis (aHR, 3.005; 95% CI, 2.503-3.607; *P* < .001), having hemiplegia (aHR, 1.430; 95% CI, 1.085-1.884; *P* = .01), having moderate or severe renal disease (aHR, 2.054; 95% CI, 1.643-2.568; *P* < .001), having leukemia (aHR, 4.541; 95% CI, 1.132-8.207; *P* = .03), and having non-HNSCC metastatic solid cancers (aHR, 1.457; 95% CI, 1.292-1.644; *P* < .001) were significant risk factors for 90-day mortality. Risk scores were categorized as very low risk (score of 0), low risk (score 1-3), moderate risk (score 4-6), and high risk (score ≥7), with 90-day mortality rates of 3.37%, 5.00% to 10.98%, 16.15% to 29.13%, and 33.93% to 37.50%, respectively. Mortality rates for patients with the same risk score in the training and test data sets were similar (score of 0, 3.27% vs 3.66%; score of 6, 27.42% vs 25.00%).

**Conclusions and Relevance:**

In this prognostic study, a 90-day mortality scoring system accurately predicted 90-day mortality among patients with locally advanced HNSCC who completed CCRT.

## Key Points

**Question:**

Can a scoring system accurately predict 90-day mortality among patients with locally advanced head and neck squamous cell carcinoma (HNSCC) after the completion of concurrent chemoradiotherapy (CCRT)?

**Findings:**

In this prognostic study of 16 029 patients with locally advanced HNSCC, a scoring system accurately predicted 90-day mortality after completion of CCRT.

**Meaning:**

More conservative treatments than CCRT could be considered for patients with locally advanced HNSCC and moderate to high risk according to this scoring system.

## Teaser

This prognostic study validates the accuracy of a predictive scoring system for 90-day mortality among patients with locally advanced head and neck squamous cell carcinoma (HNSCC) who have completed concurrent chemoradiotherapy.

## Introduction

In Taiwan, the annual incidence of head and neck squamous cell carcinoma (HNSCC) is approximately 7800 cases, and HNSCC is the sixth leading cause of cancer death in the country.^1,2,3,4^ Radiotherapy (RT) or concurrent chemoradiotherapy (CCRT) treatment for HNSCC can cause severe treatment-related toxic effects. According to the Taiwan Health Promotion Administration, the 90-day mortality of patients with locally advanced HNSCC after completion of CCRT is high in Taiwan.^4^ Thus, it is imperative to carefully select patients with locally advanced HNSCC to undergo aggressive CCRT to prevent medical waste and excessive or invalid treatment administration.

The 90-day mortality rate after completion of RT is a proposed measure for improving cancer outcomes and reducing avoidable treatment-related toxic effects.^5,6,7^ In the United Kingdom, the 90-day mortality rate among patients with HNSCC after completion of RT (3.6% per the Data Audit for Head and Neck Oncology^8^) has been proposed as the reference to indicate treatment suitability, reduce treatment-related mortality, and improve cancer outcomes.^7^ Similarly, the Scottish Cancer Taskforce recommended that the 90-day mortality of patients with HNSCC after treatment be considered a guideline for ensuring the quality of care and appropriate curative treatment administration.^9^ Other studies on different cancer sites have reported 90-day mortality rates of 1.7% and 4.8% after radical RT.^10,11^ However, only a few reports have documented the 90-day post-CCRT mortality rate specific to HNSCC, with a reported post-CCRT early mortality rate (ie, not specific to a 90-day threshold) of 5.4% to 18.3%.^12,13,14^ In these studies, patient parameters that contributed to early mortality after RT included older age, male sex, low socioeconomic status, elevated white blood cell count, vascular comorbidities, poor performance status, low body mass index, and low peripheral blood total lymphocyte count. These factors were associated with increased treatment-related mortality.^7,8,9,10,11,12,13,14^

All prescribed radical treatments should achieve a 90-day mortality rate of less than 5% across all disease groups.^15^ Thus, the primary aim of this study was to develop a suitable and accurate predictive scoring system for Taipei Medical University (TMU) to predict the 90-day mortality among patients with locally advanced HNSCC who are receiving CCRT. Using the TMU-CCRT Mortality Predictor Score to predict 90-day mortality, crucial information can be obtained regarding the treatment options for patients with locally advanced HNSCC.

## Methods

This study was approved by the institutional review board of Taipei Medical University; informed consent was waived because the data were from the publicly available National Health Insurance Research Database (NHIRD). This study followed the Transparent Reporting of a Multivariable Prediction Model for Individual Prognosis or Diagnosis (TRIPOD) reporting guideline.

### Database

The study cohort was selected from the Taiwan Cancer Registry database (TCRD). We conducted a population-based cohort study using the NHIRD linked to the TCRD. The TCRD was established in 1979 and contains 97% of cancer cases in Taiwan.^16^ Data in the NHIRD is obtained from the National Health Insurance Program, which includes more than 25.7 million beneficiaries. It covers more than 99% of the population in Taiwan. The NHIRD includes all medical claims data on disease diagnoses, procedures, drug prescriptions, demographic characteristics, and enrollment profiles of all beneficiaries.^17^ The NHIRD and TCRD are linked by encrypted patient identifiers. Additionally, NHIRD data are linked to the death registry to ascertain the vital status and cause of death of each patient. The TCRD of the Collaboration Center of Health Information Applications contains detailed patient information, including clinical cancer stages, RT doses, habits (ie, smoking, betel nut chewing, and alcohol consumption), surgical procedures, surgical techniques, and chemotherapy regimens.^2,3,18,19^

### Selection of Study Participants

This study assessed 16 029 patients with locally advanced HNSCC (ie, clinical stage III-IVB) who had completed CCRT between January 1, 2006, and December 31, 2015. Patients who underwent concurrent platinum-based chemotherapy at a dose of 100 mg/m^2^ at least twice and RT at a dose of at least 70 Gy were included.^20,21,22^ All included patients were older than 18 years. Patients with metastatic HNSCC were excluded. Eligible patients were divided into the following 2 groups based on their 90-day post-CCRT mortality, with the last date of CCRT as the index date: 90-day mortality and 90-day survival.

### Statistical Analysis

Basic demographic characteristics, including sex and age, were categorized. Patient age was determined per the index date. Patients were divided into 6 age groups (ie, 18-29, 30-39, 40-49, 50-59, 60-69, and ≥70 years). Variables of interest included demographic characteristics; American Joint Committee on Cancer clinical stages (ie, III or IV); use of tobacco, betel nut, and/or alcohol; and comorbidity profiles. Comorbidity profiles were derived based on previous studies and from the NHIRD or TCRD.^23,24,25^ Patients with myocardial infarction, cerebrovascular accident, transient ischemic attack, coronary artery disease with stents, ongoing cardiac ischemia, severe valve dysfunction, severe reduction of ejection fraction, sepsis, disseminated intravascular coagulation, adult respiratory distress syndrome, or end-stage renal disease were examined. Comorbid manifestations reported more than 1 year before the index date were not included to ensure relevance. Based on the main *International Statistical Classification of Diseases, Tenth Revision, Clinical Modification *(*ICD*-*10*-*CM*) diagnostic codes, comorbidities were identified based on a positive diagnosis in a single admission or in 2 or more repeated visits to outpatient departments within 1 year. The entire list of investigated comorbidities included diabetes, hypertension, pneumonia, chronic obstructive pulmonary disease–acute event, hepatitis B infection, hepatitis C infection, pacemaker implantation, myocardial infarction, cerebrovascular accident, transient ischemic attack, coronary artery disease, angina, heart valve dysfunction, end-stage renal disease, sepsis, chronic kidney disease, heart failure, disseminated intravascular coagulation, adult respiratory distress syndrome, aortic aneurysm, peripheral vascular disease, peptic ulcer disease, dementia, chronic pulmonary disease, connective tissue disease, mild liver disease, hemiplegia, moderate or severe renal disease, any non-HNSCC solid cancer, leukemia, lymphoma, moderate or severe liver disease, metastatic non-HNSCC solid cancer, previous thoracic surgery, smoking, alcohol use, betel nut use, obesity, asthma, and bowel obstruction. The χ^2^ test was used to compare demographic characteristics and comorbidities between the mortality and survival groups.

Identification of significant factors was required to construct the TMU-CCRT Predictive Scoring System for 90-day mortality among patients with locally advanced HNSCC receiving CCRT. Univariate and multivariable Cox proportional hazards models were used to determine the hazard ratio (HR) and 95% CI for each factor. The stepwise selection method was used to select all factors that significantly predicted 90-day mortality. A forward stepwise selection method was used to select variables that had significant associations with survival duration. Variables with coefficients greater than 0 or HRs greater than 1 were selected as risk factors to construct the TMU-CCRT Mortality Predictor Scoring System. Each factor contributed points to the score based on its HR. The stepwise method is a modification of the forward selection technique, in which variables already in the model do not necessarily stay in the model. During the implementation of the stepwise selection method, the same entry and removal approaches were used for the forward selection and backward elimination to assess the contribution of variables as they were added to or removed from a model. The minimum F-to-enter was used to add or remove a variable. The best model was chosen based on the information criterion. Factors with HRs of 1 or greater were considered risk factors. The risk point for each factor was defined as the greatest integer less than or equal to that factor’s HR in stepwise regression.^26^

Patients were divided into several groups according to their risk score. Patients with high risk scores were expected to have increased 90-day mortality after the completion of CCRT. Patients were randomly divided into a training data set and a test data set at a ratio of 3:1 to validate our risk scoring system. The risk score was reconstructed on training data, and the score on test data was computed (eTable 1 in the Supplement). Furthermore, to validate the scoring system’s discrimination ability, we randomly split the data into training and test parts and compared the 90-day mortality rate in each stratum (eTable 1 in the Supplement). The similarity of mortality rates assessed the scoring system’s predictivity. Because area under the receiver operating characteristic curve is a measure for discriminant analyses, such as the logistic model or Gaussian latent dirichlet allocation and not the Cox model, we have not reported the area under the receiver operating characteristic curve value. Finally, the mortality rates of patients with the same risk scores in the training and test data sets were compared to assess the system’s accuracy. The long-term mortality rate of the TMU-CCRT Mortality Predictor Scoring System was also estimated using the Kaplan-Meier method, and the differences among the risk groups were determined using the log-rank test. All statistical analyses were performed using SAS for Windows version 9.2 (SAS Institute). A 2-tailed *P* < .05 was considered statistically significant.

## Results

Table 1 provides the comparative statistical description of basic information and comorbidity profiles of patients in the 90-day mortality and 90-day survival groups. Of the 16 029 patients examined, 1068 (1016 [95.1%] men; mean [SD] age, 55.11 [11.45] years) died before reaching the 90-day threshold, whereas 14 961 (14 080 [94.1%] men; mean [SD] age, 52.07 [9.99] years) survived. Thus, CCRT for locally advanced HNSCC may achieve an overall 90-day mortality rate of 6.66%. Mean (SD) age (55.11 [11.45] years vs 52.07 [9.99] years; *P* < .001) and the frequencies of some comorbidities (eg, pneumonia, 180 [16.9%] vs 933 [6.2%]; *P* < .001; chronic obstructive pulmonary disease–acute event, 100 [9.4%] vs 928 [6.2%]; *P* < .001; myocardial infarction, cerebrovascular accident, transient ischemic attack, or coronary artery disease, 143 [13.4%] vs 1404 [9.4%]; *P* < .001) differed significantly between the mortality and survival groups (Table 1). By contrast, no significant intergroup differences were observed for sex, alcohol consumption, betel nut chewing, clinical stage, or cigarette smoking.

**Table 1.** Demographic Characteristics of 90-Day Mortality and 90-Day Survival Groups

Characteristic	No. (%)		*P* value
	Mortality group (n = 1068)	Survival group (n = 14 961)	
Age, mean (SD), y	55.11 (11.45)	52.07 (9.99)	<.001
Sex			
Women	52 (4.9)	881 (5.9)	.19
Men	1016 (95.1)	14 080 (94.1)	
AJCC clinical stage			
III	374 (35.0)	5237 (35.0)	.97
IV	694 (65.0)	9724 (65.0)	
Cigarette smoking	982 (92.0)	13 764 (92.0)	.97
Betel nut chewing	1001 (93.7)	14 063 (94.0)	.99
Alcohol use	747 (70.0)	10 470 (70.0)	.91
Comorbidities			
Diabetes	46 (4.3)	513 (3.4)	.15
Hypertension	255 (23.9)	3584 (24.0)	.98
Pneumonia	180 (16.9)	933 (6.2)	<.001
COPD-AE	100 (9.4)	928 (6.2)	<.001
Hepatitis B infection	15 (1.4)	313 (2.1)	.16
Hepatitis C infection	24 (2.2)	373 (2.5)	.69
Implanted pacemaker	1 (0.1)	6 (<0.1)	.96
MI, CVA, TIA, or CAD	143 (13.4)	1404 (9.4)	<.001
Heart valve dysfunction	14 (1.3)	146 (1.0)	.37
Sepsis	160 (15.0)	549 (3.7)	<.001
Chronic kidney disease	89 (8.3)	446 (3.0)	<.001
Heart failure	29 (2.7)	207 (1.4)	.001
adult respiratory distress syndrome	1 (0.1)	3 (<0.1)	.64
Aortic aneurysm	2 (0.2)	15 (0.1)	.72
PVD	17 (1.6)	218 (1.5)	.82
PUD	161 (15.1)	2042 (13.6)	.21
Dementia	44 (4.1)	376 (2.5)	.002
Chronic pulmonary disease	106 (9.9)	992 (6.6)	<.001
Connective tissue disease	12 (1.1)	131 (0.9)	.51
Mild liver disease	189 (17.7)	2463 (16.5)	.32
Hemiplegia	57 (5.3)	437 (2.9)	<.001
Moderate or severe renal disease	91 (8.5)	471 (3.1)	<.001
Other solid cancers	28 (2.6)	260 (1.7)	.048
Leukemia	2 (0.2)	5 (<0.1)	.12
Lymphoma	4 (0.4)	46 (0.3)	.92
Moderate or severe liver disease	83 (7.8)	1069 (7.1)	.48
Other metastatic solid cancers	549 (51.4)	6244 (41.7)	<.001

Abbreviations: AJCC, American Joint Committee on Cancer; CAD, coronary artery disease; COPD-AE, chronic obstructive pulmonary disease–acute episode; CVA, cerebrovascular accident; MI, myocardial infarction; PUD, peptic ulcer disease; PVD, peripheral vascular disease; TIA, transient ischemic attack.

The results of the regression analysis for univariate and multivariable Cox proportional hazards models are presented in eTable 2 in the Supplement and Table 2 and Table 3, respectively. Because of collinearity between the factors, fewer factors were observed to be significant in the multivariable model than in the univariate model. For example, age 60 years and older (compared with age younger than 60 years) was significant in the univariate model (adjusted HR [aHR], 1.112; 95% CI, 1.034-1.324; P = .03) but not in the multivariate model (Table 2). Thus, we applied the stepwise method in the multivariable model for variable selection. Table 3 presents all the significant factors, such as age 50 years or older (aHR, 1.263; 95% CI, 1.104-1.445; *P* < .001), age 70 years or older (aHR, 2.183; 95% CI, 1.801-2.645; *P* < .001), pneumonia (aHR, 1.946; 95% CI, 1.636-2.314; *P* < .001), sepsis (aHR, 3.005; 95% CI, 2.503-3.607; *P* < .001), hemiplegia (aHR, 1.430; 95% CI, 1.085-1.884; *P* = .01), moderate or severe renal disease (aHR, 2.054; 95% CI, 1.643-2.568; *P* < .001), leukemia (aHR, 4.541; 95% CI, 1.132-8.207; *P* = .03), and non-HNSCC metastatic solid tumor (aHR, 1.457; 95% CI, 1.292-1.644; *P* < .001).

**Table 2.** All-Cause 90-Day Mortality Risk Assessment Using a Multivariate Cox Proportional Hazards Model

Factor	aHR (95% CI)^a^	*P* value
Age, y		
≥30, compared with <30	1.038 (0.425-1.107)	.44
≥40, compared with <40	1.207 (0.924-1.579)	.16
≥50, compared with <50	1.176 (1.008-1.373)	.04
≥60, compared with <60	1.112 (1.034-1.324)	.03
≥70, compared with <70	2.021 (1.617-2.526)	<.001
Sex		
Men, compared with women	1.283 (0.966-1.703)	.09
AJCC clinical stage		
IV, compared with III	1.132 (0.687-1.890)	.67
Cigarette smoking	1.149 (0.536-1.218)	.55
Betel nut chewing	1.318 (0.935-1.468)	.28
Alcohol use	1.401 (0.945-1.435)	.34
Comorbidities		
Diabetes	0.995 (0.736-1.345)	.97
Hypertension	0.75 (0.641-1.877)	.50
Pneumonia	1.867 (1.567-2.225)	<.001
COPD-AE	0.951 (0.687-1.315)	.76
Hepatitis B infection	0.731 (0.435-1.229)	.24
Hepatitis C infection	0.736 (0.463-1.168)	.19
Implanted pacemaker	0.996 (0.137-7.262)	>.99
MI, CVA, TIA, or CAD	1.117 (0.883-1.413)	.36
Heart valve dysfunction	0.983 (0.572-1.690)	.95
Sepsis	2.972 (2.473-3.571)	<.001
Chronic kidney disease	1.280 (0.641-2.556)	.48
Heart failure	1.262 (0.856-1.861)	.24
Disseminated intravascular coagulation	4.251 (0.589-7.673)	.15
Adult respiratory distress syndrome	4.388 (0.611-8.533)	.14
Aortic aneurysm	1.158 (0.284-4.725)	.84
PUD	0.839 (0.483-1.459)	.54
PVD	0.956 (0.804-1.136)	.61
Dementia	1.296 (0.948-1.772)	.10
Chronic pulmonary disease	1.199 (0.877-1.64)	.26
Connective tissue disease	1.114 (0.628-1.976)	.71
Mild liver disease	1.003 (0.848-1.187)	.97
Hemiplegia	1.253 (1.194-1.756)	.02
Moderate or severe renal disease	2.579 (1.796-3.134)	<.001
Other solid cancers	1.159 (0.794-1.690)	.44
Leukemia	4.910 (1.218-6.796)	.03
Lymphoma	1.076 (0.402-2.882)	.88
Moderate or severe liver disease	1.164 (0.893-1.518)	.26
Other metastatic solid cancers	1.472 (1.304-1.661)	<.001

Abbreviations: aHR, adjusted hazard ratio; AJCC, American Joint Committee on Cancer; CAD, coronary artery disease; COPD-AE, chronic obstructive pulmonary disease–acute event; CVA, cerebrovascular accident; MI, myocardial infarction; PUD, peptic ulcer disease; PVD, peripheral vascular disease; TIA, transient ischemic attack.

^a^ All variables were used in multivariate analysis.

**Table 3.** Stepwise Selection of the Multivariable Cox Proportional Hazards Model for All-Cause 90-Day Mortality

Factor	aHR (95% CI)^a^	*P* value	Assigned points
Age, y			
≥50, compared with <50	1.263 (1.104-1.445)	<.001	1
≥70, compared with <70	2.183 (1.801-2.645)	<.001	2
Comorbidities			
Pneumonia	1.946 (1.636-2.314)	<.001	1
Sepsis	3.005 (2.503-3.607)	<.001	3
Hemiplegia	1.430 (1.085-1.884)	.01	1
Moderate or severe renal disease	2.054 (1.643-2.568)	<.001	2
Leukemia	4.541 (1.132-8.207)	.03	4
Other metastatic solid cancers	1.457 (1.292-1.644)	<.001	1

Abbreviation: aHR, adjusted hazard ratio.

^a^ All variables were used in multivariate analysis.

The risk scores were calculated based on the risk factors and corresponding risk points based on our previous study.^26^ The risk points for these factors are displayed in Table 3, and risk score distribution is presented in Table 4. According to our model, a patient with a risk score of 0 would have a 90-day mortality rate of 3.37%, whereas a patient with a risk score of 9 would have a 90-day mortality rate of 37.50%. Our data indicated that higher risk scores corresponded to higher mortality risk. Therefore, to quantify our results, the risk scores of 0, 1 to 3, 4 to 6, and 7 or greater were categorized to indicate very low, low, moderate, and high risk, respectively (with 90-day mortality rates of 3.37%, 5.00%-10.98%, 16.15%-29.13%, and 33.93%-37.50%, respectively) (Table 4). The mortality rates for patients with the same risk score in the training and test data sets were similar (score of 0, 3.27% vs 3.66%; score of 6, 27.42% vs 25.00%), thereby indicating that our risk scoring system might have an excellent prediction accuracy for test data (eTable 1 in the Supplement).

**Table 4.** All-Cause 90-Day Mortality Assessment Using the Mortality Predictor Scoring System

Cumulative score	No.		90-d mortality rate after CCRT, %
	Survivors	Deaths	
0	3210	112	3.37
1	6577	346	5.00
2	3278	243	6.90
3	916	113	10.98
4	597	115	16.15
5	233	74	24.10
6	90	37	29.13
7	37	19	33.93
8	18	6	25.00
≥9	5	3	37.50
Total	14 961	1068	6.66

Abbreviation: CCRT, concurrent chemoradiotherapy.

The 90-day mortality rate and 5-year overall survival of the patients were estimated using the Kaplan-Meier method to analyze the risk of mortality associated with the 4 risk strata (Figure; eFigure in the Supplement). Our analysis revealed that the 90-day survival rates were 96.63% for the very low–risk group, 93.41% for the low-risk group, 80.28% for the moderate-risk group, and 68.18% for the high-risk group, respectively (*P* < .001) (Figure), whereas 5-year overall survival rates were 58.75% for the very low–risk group, 56.11% for the low-risk group, 31.79% for the moderate-risk group, and 21.15% for the high-risk group (*P* < .001) (eFigure in the Supplement).

**Figure.**
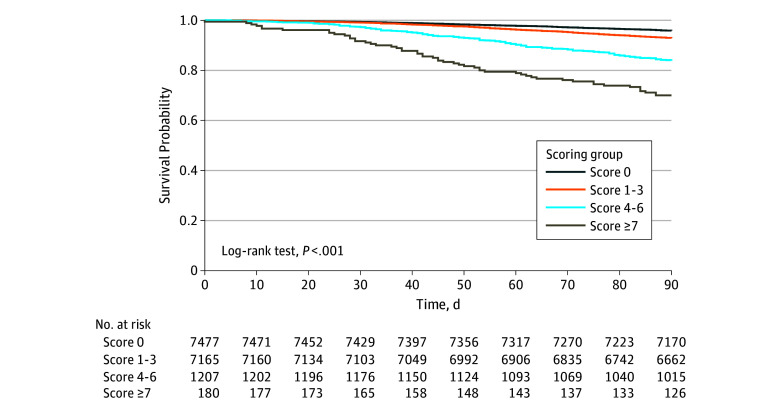
Kaplan-Meier Survival Curve for 90-Day Mortality for the 4 Risk Groups

## Discussion

Based on previous randomized clinical trial results,^20,21,22,27^ CCRT is considered the conventional treatment for unresectable locally advanced HNSCC. However, these studies have not investigated relevant factors, such as poor performance status, severe underlying diseases, and older age.^20,21,22,27^ Indeed, the 90-day mortality rate among patients with locally advanced HNSCC following CCRT may be because of toxic iatrogenic manifestations. Common clinical etiologies of CCRT-related death in locally advanced HNSCC include hepatitis flare-ups, respiratory diseases, metabolic diseases, fatal hemorrhage, acute renal failure, or iatrogenic immunocompromised conditions with aspiration pneumonia, cardiac death, or sepsis.^5,28,29,30^ Given the clinical evidence and study limitations, we reasoned that prescribing aggressive CCRT to all patients with locally advanced HNSCC, including those with severe underlying diseases, might be inappropriate and dangerous because of the increased risk of treatment-related toxic effects.^5,6,24,31^ Notably, age and comorbid diseases are 2 independent prognostic factors strongly associated with survival, mainly because of an increase in noncancer-related mortality and severe underlying diseases.^31^ However, current data regarding the differences in cancer-specific survival in various comorbid profiles as well as between young and elderly patients are scarce.^31^ In addition, the suitability of active surveillance or RT alone as less toxic therapeutic alternatives to the commonly prescribed CCRT in some specific patients with locally advanced HNSCC has not been addressed, to our knowledge.^32^

Our findings indicated that elderly patients with locally advanced HNSCC undergoing CCRT were more likely to die within 90 days of therapy completion than younger patients. The 90-day mortality rate among elderly patients with HNSCC receiving RT using our risk score was slightly higher than that obtained in the study by Hamilton et al.^5^ Notably, CCRT use was less than 30% in the study by Hamilton et al,^5^ and 31% of patients had early-stage HNSCC. In the study by Hamilton et al,^5^ the interventions were mainly RT alone (among >70% of patients), not CCRT, for all stages of HNSCC. In the present study, all patients with HNSCC had advanced-stage disease (ie, stage III-IV) and were receiving definitive CCRT. Our study had no patients with early-stage HNSCC or patients who were receiving RT alone. Therefore, the 90-day mortality that Hamilton et al^5^ found is different from our study. Our results further indicated that comorbidities, including myocardial infarction, cerebrovascular accident, transient ischemic attack, coronary artery disease, pneumonia, chronic obstructive pulmonary disease–acute event, sepsis, chronic kidney disease, heart failure, dementia, chronic pulmonary disease, hemiplegia, moderate or severe renal disease, other non-HNSCC solid cancers, and other non-HNSCC metastatic solid cancers, significantly increased the risk of CCRT-related mortality within 90 days of treatment completion. However, further investigation is warranted to determine whether the observed mortality was directly related to CCRT or the underlying HNSCC and comorbid illnesses. Nonetheless, our data strongly indicated that elderly patients with locally advanced HNSCC and patients with locally advanced HNSCC and severe underlying diseases had poor tolerance to CCRT. This poor tolerance was also associated with compromised adherence of patients, often resulting in the prolonged prescription of CCRT. Concordant with our findings, some studies have reported that both treatment effectiveness and patient adherence are compromised in patients with cancer and comorbidities.^3,33^

Our data revealed that being aged 50 years or older and 70 years or older as well as having pneumonia, sepsis, hemiplegia, moderate or severe renal disease, leukemia, and non-HNSCC metastatic solid tumor were significant risk factors (Table 2, Table 3, and Table 4). Indeed, previous studies have suggested that age, pneumonia, sepsis, hemiplegia, moderate or severe renal disease, presence of other cancers, and non-HNSCC metastatic cancers are independent poor prognostic factors of overall mortality risk in patients with HNSCC receiving curative treatments.^5,6,24,34,35,36,37,38^ The major causes of 90-day mortality in patients with HNSCC were aspiration pneumonia, infections, and sepsis.^39,40,41,42,43^ The mechanisms of aspiration pneumonia, infections, and sepsis were most likely from chemotherapy-related immunocompromised sepsis, RT-related poor swallowing–induced aspiration pneumonia, and comorbidity-related poor self-care ability.^39,40,41,42,43,44^ Self-care ability in hemiplegia patients was poor, with a higher rate of aspiration pneumonia compared with physically able patients with moderate or severe liver disease.^45^ Leukemia and lymphoma require different treatments. The type of leukemia or lymphoma may also make a difference in how cancer is treated. The survival rate refers to the specific types of leukemia or lymphoma and may vary by stage of cancer at diagnosis.^46,47,48,49,50,51^ The most common type of lymphoma in Taiwan is diffuse large B-cell lymphoma, and the most common leukemia in Taiwan is acute lymphoblastic leukemia.^52,53^ More leukemia patients receive long-term and intensive chemotherapy or stem cell transplantation with long-term immunosuppressive drug use than lymphoma patients.^54,55,56,57,58^ Therefore, the 90-day mortality is higher among patients with HNSCC receiving CCRT who had previous leukemia because patients with leukemia would have more underlying treatment-related toxic effects and immunocompromised conditions when they receive standard aggressive CCRT for their newly diagnosed locally advanced HNSCC.^54,55,56,57,58^ In the current study, our 90-day post-CCRT mortality scoring system accurately predicted not only the mortality in the first 90 days after CCRT but also the long-term survival in patients with locally advanced HNSCC after CCRT. Regarding overall survival, risk factors identified in the current study were concordant with those identified in previous studies.^34,35,36,37,38^

Fatal adverse events have been reported in less than 5% of patients with locally advanced HNSCC who have undergone CCRT treatment.^6,12^ However, compared with RT treatment, CCRT treatment is associated with higher mortality among elderly patients with HNSCC and multiple underlying diseases.^5,6,24,25^ Treatment-related mortality in cancer patients was reported to be between 5% and 15%.^5,6,59,60^ Because the CCRT-related mortality was greater than 15% among some of our patients with locally advanced HNSCC (a result likely unacceptable for the HNSCC tumor board),^5,6,59,60^ alternative treatments, such as RT alone or chemotherapy alone, should be considered. Our findings suggested that aggressive CCRT was most likely unsuitable for patients with locally advanced HNSCC with a moderate and high risk according to our classification. Therefore, we believe that RT alone or chemotherapy alone are better options for these patients. Nonetheless, future studies are required to confirm this.

We wanted to determine whether the 90-day mortality score was a suitable tool for estimating 5-year overall survival. If the score is effective in predicting the long-term overall survival, then we can use the scores to predict not only the 90-day mortality rate but also long-term survival. The predictive score of mortality could be used for survival adjustment or treatment information in future trials and definitive CCRT. The shorter survival in high-risk predictive scores might reflect older age, more comorbidities, or higher intolerance of treatment-related complications and toxic effects, such that locally advanced HNSCC patients with high risk scores also had higher 5-year mortality rates.

To our knowledge, our study was the first to consider age and comorbidity profiles in the prediction of 90-day post-CCRT mortality in patients with locally advanced HNSCC. Consistent with our predictions, both factors exhibited significant differences among the various risk strata. Our findings suggested that the TMU-CCRT Mortality Predictor Scoring System was a valid and specific system that could accurately predict overall mortality among patients with locally advanced HNSCC in the first 90 days after treatment as well as in the long term.

Our new scoring system accurately predicted the 90-day mortality in patients with locally advanced HNSCC who completed CCRT. We demonstrated that this predictive scoring system for CCRT-related mortality in patients with locally advanced HNSCC can be used as a reference to make therapeutic decisions. Thus, our predictive 90-day post-CCRT mortality scoring system for patients with locally advanced HNSCC could be a valuable tool for physicians to assess the physical status of patients and make appropriate therapeutic decisions.

### Limitations

This study has limitations. First, CCRT-induced toxic effects were not objectively measured or quantified. Thus, estimates of CCRT-related mortality in patients with HNSSC might have been prone to error. However, the study design included factors such as the tolerance of treatment-related toxic effects in old age and comorbidities. Most deaths might be related to old age and comorbidities, which render a patient unable to tolerate the toxic effects of aggressive CCRT. Second, because all patients with locally advanced HNSCC enrolled in this study had Asian race, the extrapolation of our findings to non-Asian populations may not be entirely suitable. However, considering that treatment for locally advanced HNSCC does not differ between Western and Asian countries,^61^ the predictive scores are still clinically pertinent as a pretreatment reference for CCRT in patients with locally advanced HNSCC, irrespective of race/ethnicity. Third, the diagnoses of all comorbid conditions were based on *ICD*-*9*-*CM* codes. Considering that the Taiwan Cancer Registry Administration randomly reviews medical records, interviews patients, and ensures that hospitals with outlier charges or practices are audited and subsequently heavily penalized if malpractice or discrepancies are identified, we believe diagnoses to be accurate. In addition, previous studies have verified and proven the quality and precision of *ICD*-*9*-*CM* codes in Taiwan.^62,63^ Therefore, we believe the conclusion of the study would not be overturned. Nevertheless, to obtain accurate information on population specificity and disease occurrence, a large-scale randomized clinical trial that carefully compares selected elderly patients with multiple comorbidities who have received suitable treatments is required. Fourth, we have split the sample randomly, which is not as rigorous a test as validation in a completely different data set. Extrapolation of our risk groups to another data set may not be entirely suitable. However, we reported the results of univariate and multivariable Cox models on the entire data set, and the validation procedure was used to assess the out-sample performance of our method. When we performed validation using training and test data, the model was refitted only on training data (ie, the parameter estimation was different from estimation on entire data set reported before), and the fitted model was used on the test data. If we only reported the results on training data, we would lose substantial amounts of information on test data. Therefore, the current order of 2 parts was ideal for describing the results. Fifth, the risk of 90-day mortality should be a part of any conversation between patients and physicians. Whether aggressive CCRT is appropriate would probably depend on the results of a future randomized clinical trial comparing CCRT with weekly cisplatin, RT alone, or watchful waiting. Locally advanced HNSCC might receive a nonstandard CCRT regimen after analysis of the TMU-CCRT Mortality Predictor Scoring System. However, our analysis likely underestimated the morbidity and mortality associated with aggressive CCRT because we limited the data set to patients who were sufficiently healthy to tolerate at least 2 doses of platinum-based chemotherapy. By excluding patients who were recommended for CCRT treatment but were only able to receive 1 dose of bolus platinum, were treated with a lower-dose (eg, weekly) platinum, or were unable to receive the full prescribed dose of RT, we excluded the patients with the most severe illness. Sixth, although informative, the TCRD lacks information regarding dietary habits, socioeconomic status, or body mass index, all of which may be potential risk factors for mortality in the context of CCRT.

## Conclusions

This study’s findings suggest that the TMU-CCRT Mortality Predictor Scoring System can accurately predict the 90-day mortality in patients with locally advanced HNSCC who completed CCRT. Based on our results, alternative non-CCRT treatments should be considered in patients with locally advanced HNSCC and moderate or high risk.

## Notes

Supplement. **eTable 1. **Validation of TMU-CCRT Mortality Predictor Score **eTable 2. **All-Cause 90-Day Mortality Risk Assessment by Using a Univariate Cox Proportional Hazards Model **eFigure. **Kaplan-Meier Plot of 5-Year Overall Survival for the 4 Score Groups
