# Supplementary material for: Assessment of Predictive Scoring System for 90-Day Mortality Among Patients With Locally Advanced Head and Neck Squamous Cell Carcinoma Who Have Completed Concurrent Chemoradiotherapy
Source: JAMA Netw Open. 2020 Mar 26;3(3):e1920671. doi: 10.1001/jamanetworkopen.2019.20671 (PMC12507455; doi:10.1001/jamanetworkopen.2019.20671)
Supplement: Supplement. — eTable 1. Validation of TMU-CCRT Mortality Predictor Score eTable 2. All-Cause 90-Day Mortality Risk Assessment by Using a Univariate Cox Proportional Hazards Model eFigure. Kaplan-Meier Plot of 5-Year Overall Survival for the 4 Score Groups [file jamanetwopen-e1920671-s001.pdf]

Supplementary Online Content

Lin K-C, Chen T-M, Yuan KS-P, Wu ATH, Wu S-Y. Assessment of predictive scoring system for 90-day mortality among patients with locally advanced head and neck squamous cell carcinoma who have completed concurrent chemoradiotherapy. *JAMA Netw Open*. 2020;3(3):e1920671. doi:10.1001/jamanetworkopen.2019.20671

eTable 1. Validation of TMU-CCRT Mortality Predictor Score
eTable 2. All-Cause 90-Day Mortality Risk Assessment by Using a Univariate Cox Proportional Hazards Model
eFigure. Kaplan-Meier Plot of 5-Year Overall Survival for the 4 Score Groups

This supplementary material has been provided by the authors to give readers additional information about their work.

© 2020 Lin K-C et al. *JAMA Network Open.*

eTable 1. Validation of TMU-CCRT Mortality Predictor Score

	Training Data			Test Data (randomly selection from database)		
Scores	No. of survivors	No. of deaths	Mortality rate	No. of survivors	No. of deaths	90-day Mortality rate after CCRT
0	2367	80	3.27%	843	32	3.66%
1	4942	263	5.05%	1635	83	4.83%
2	2546	186	6.81%	820	65	7.34%
3	756	121	13.80%	254	31	10.88%
4	442	93	17.38%	130	28	17.72%
5	102	33	24.44%	35	16	31.37%
6	45	17	27.42%	18	6	25.00%
7	16	10	38.46%	4	0	0.00%
8+	6	4	40.00%	0	0	--
Total	11222	807	6.71%	3739	261	6.53%

© 2020 Lin K-C et al. *JAMA Network Open.*

eTable 2. All-Cause 90-Day Mortality Risk Assessment by Using a Univariate Cox Proportional Hazards Model

Predictor Factor	Crude HR	95% CI	*p* value
Age (years)			
≥ 30 (REF: < 30 y/o)	1.495	1.17-1.911	.0013
≥ 40 (REF: < 40 y/o)	1.415	1.247-1.607	<.0001
≥ 50 (REF: < 50 y/o)	1.585	1.393-1.804	<.0001
≥ 60 (REF: < 60 y/o)	2.537	1.517-2.841	<.0001
≥70 (REF: < 70 y/o)	2.614	1.919-3.604	<.0001
Sex = Male (REF: female)	2.784	0.696-5.129	.1477
AJCC clinical stage IV (REF: stage III)	1.438	1.356-1.829	<.0001
Cigarette smoking habitus (REF: Non-user)	1.162	0.937-1.679	.0613
Betel nut chewing habitus (REF: Non-user)	1.061	0.947-1.374	.0768
Alcohol use (REF: Non-user)	1.141	0.959-1.460	.0822
Comorbidities (REF: No comorbidity)			
DM	1.267	0.943-1.702	.1163
HTN	0.997	0.866-1.147	.9621
Pneumonia	2.884	2.457-3.385	<.0001
COPD	1.537	1.251-1.889	<.0001
Hepatitis B	0.672	0.404-1.119	.1265
Hepatitis C	0.898	0.599-1.345	.6004
Implanted pacemaker	2.299	0.325-6.273	.4044

© 2020 Lin K-C et al. *JAMA Network Open.*

MI, CVA, TIA, or CAD	1.478	1.24-1.763	<.0001
Heart valve dysfunction	1.349	0.796-2.285	.2655
Sepsis	4.238	3.582-5.013	<.0001
CKD	2.805	2.258-3.484	<.0001
Heart failure	1.93	1.335-2.792	.0005
DIC	5.981	0.845-6.325	.0732
ARDS	4.055	0.571-8.808	.1616
aortic aneurysm	1.87	0.467-7.484	.3765
PAD	1.115	0.645-1.927	.6956
PVD	1.122	0.949-1.327	.1777
Dementia	1.638	1.212-2.215	.0013
Chronic pulmonary disease	1.526	1.249-1.865	<.0001
Connective tissue disease	1.262	0.715-2.230	.4221
Mild liver disease	1.088	0.930-1.273	.2928
Hemiplegia	1.838	1.408-2.400	<.0001
Moderate or severe renal disease	2.73	2.202-3.384	<.0001
Any other solid cancers	1.492	1.025-2.171	.0368
Leukemia	5.200	1.300-8.900	.0197
Lymphoma	1.212	0.454-3.235	.7011
Moderate or severe liver disease	1.09	0.871-1.364	.4509
Other Metastatic solid cancers	1.453	1.289-1.638	<.0001

Diabetes mellitus, DM; hypertension, HTN; Chronic Obstructive Pulmonary Disease, COPD; Hepatitis B, HBV; Hepatitis C, HCV; myocardial infarction, MI; cerebral

© 2020 Lin K-C et al. *JAMA Network Open.*

vascular accident, CVA; transient ischemic attack, TIA; coronal arterial disease, CAD; end stage renal disease, ESRD; Chronic kidney disease, CKD; disseminated intravascular coagulation, DIC; adult respiratory distress syndrome, ARDS; peripheral vascular disease, PVD; peptic ulcer disease, PVD.

© 2020 Lin K-C et al. *JAMA Network Open.*

eFigure. Kaplan-Meier Plot of 5-Year Overall Survival for the 4 Score Groups

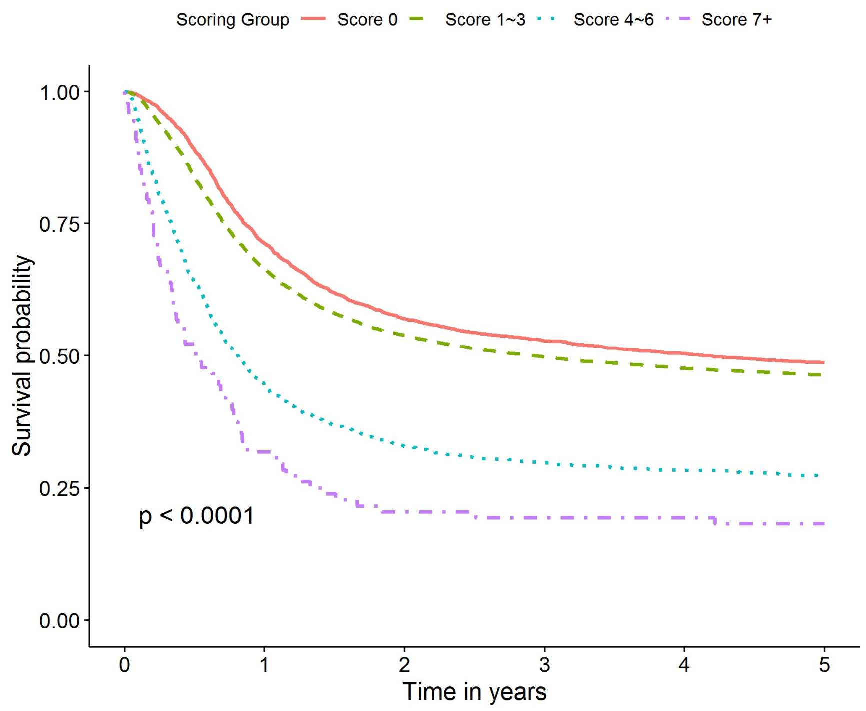

© 2020 Lin K-C et al. *JAMA Network Open.*
